# Determination of the Lewis acidity of amide–AlCl_3_ based ionic liquid analogues by combined *in situ* IR titration and NMR methods

**DOI:** 10.1039/c8ra01845f

**Published:** 2018-04-10

**Authors:** Pengcheng Hu, Wei Jiang, Lijuan Zhong, Shu-Feng Zhou

**Affiliations:** College of Chemical Engineering, Huaqiao University Xiamen 361021 Fujian China hupc1987@hqu.edu.cn szhou@hqu.edu.cn

## Abstract

A combinatorial method to determine both acidic strength and acidic amount of each Lewis acid site in amide–AlCl_3_ based ionic liquid (IL) analogues was developed by the combination of *in situ* IR titration and NMR analysis. ^31^P NMR was used to distinguish effectively the acidic strength of each Lewis acid site in the amide–AlCl_3_ based IL analogues. Nitrobenzene was used as a molecular probe to measure the total Lewis acidic amount of the amide–AlCl_3_ based IL analogues by *in situ* IR titration. The acidic amount of each Lewis acid site in the amide–AlCl_3_ based IL analogues was calculated with the assistance of ^27^Al NMR analysis.

## Abstract 2

The Lewis acidic strength and amount of amide–AlCl_3_ IL analogues are determined by the combination of *in situ* IR titration and NMR analysis.

## Graphical abstract

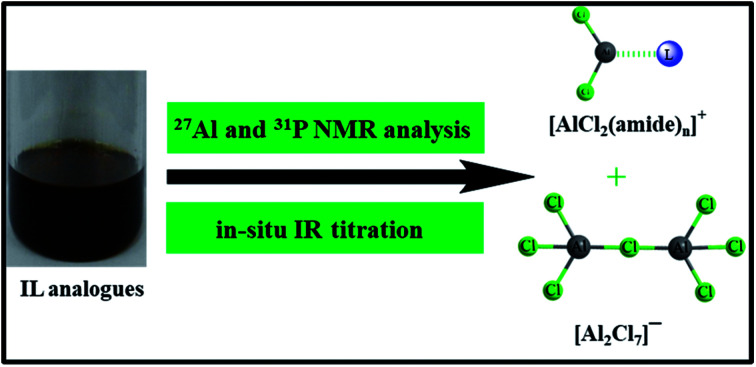

Acidic amide–AlCl_3_ based ionic liquid (IL) analogues have attracted significant attention as good alternatives to traditional imidazolium and pyridinium based halometallate ILs due to their broad acidity-adjusting range, high catalytic activity, low toxicity and cost, and easy preparation.^1–3^ Amide–AlCl_3_ based IL analogues exhibit a mixture of neutral molecular Al species, and cationic and anionic Al species in equilibrium, which contribute to the incomplete asymmetric splitting of Al_2_Cl_6_ under the induction of amide.^4,5^ Therefore, multiple Lewis acidic species with catalytic activity exist in these ILs analogues.^6^ The Lewis acidity of the amide–AlCl_3_ based IL analogues, including acidic strength and amount, is correlated with their catalytic activity and selectivity.^7–9^ Hence, it is necessary to establish a suitable method to determine the acidic strength and amount of each Lewis acid in these IL analogues, which can guide corresponding acid-catalyzed reactions.

For traditional ILs, the spectral measurement methods of the acidity are mainly UV-vis, NMR and IR spectroscopies. The UV-vis spectroscopy method determines semi-quantitatively the acidic strength of total Brønsted acid in ILs according to the Hammett function,^10–12^ but it could not be applied in the analysis of Lewis acid in ILs, such as [Al_2_Cl_7_]^−^ in chloroaluminate ILs. The Lewis acidic strength can be quantified by the Gutmann acceptor number, which is directly proportional to the ^31^P NMR chemical shift of triethylphosphineoxide (TEPO) dissolved in ILs.^13,14^ The ^31^P NMR method can distinguish effectively the acidic strength of each Lewis acid in ILs with multiple Lewis acids, but it could not measure the acidic amount of each Lewis acid.^15–17^ The traditional KBr tabletting IR uses nitrogen-containing compounds as molecular probes, such as pyridine and ethanenitrile. The change in the IR frequencies of the molecular probes is correlated to the acidic strength of the acid species in ILs. The tabletting IR method can distinguish evidently the Brønsted and Lewis acid according to the wavenumber of the characteristic peaks.^18,19^ For example, two peak at 1450 cm^−1^ and 1540 cm^−1^ were the indication of pyridine coordinated to Lewis and Brønsted acid, respectively.^20^ But this method neither distinguishes easily the acidic strength of each Lewis acid in ILs with multiple Lewis acids because of the overlap of characteristic peaks, nor can it measure the acidic amount of each Lewis acid. In addition, infrared studies of ammonia adsorption and microcalorimetry were also used by Dupont Company to investigate the acidity of zeolite.^21^

In this communication, we first establish a combinatorial method to determine the acidity of amide–AlCl_3_ based IL analogues with multiple Lewis acids by combining *in situ* IR titration with NMR analysis. This method not only distinguished effectively the acidic strength of each Lewis acid in amide–AlCl_3_ based IL analogues, but also measured the acidic amount of each Lewis acid.

Firstly, ^31^P NMR was used to identify the acidic strength of each Lewis acid in amide–AlCl_3_ based IL analogues, as shown in Fig. 1. A single peak at 83.48 ppm was observed in the ^31^P NMR spectra of molecular probe (TEPO) dissolved in neat Et_3_NHCl–AlCl_3_ IL (molar ratio of Et_3_NHCl to AlCl_3_ was 0.65), which was assigned to the coordination of TEPO to Lewis acid. This result indicated that neat Et_3_NHCl–AlCl_3_ IL only contained single Lewis acid, namely [Al_2_Cl_7_]^−^. However, two peaks at 83.48 and 84.92 ppm were observed in neat NMA–AlCl_3_ IL analogue (molar ratio of *N*-methylacetamide to AlCl_3_ was 0.65, marked as 0.65NMA–1.0AlCl_3_) with the addition of TEPO. This phenomenon indicated that another Lewis acid in addition to [Al_2_Cl_7_]^−^ existed in 0.65NMA–1.0AlCl_3_. The peak at 84.92 ppm was assigned to the cationic Al species because the molecule Al species was neutral.^5^ Meanwhile, the acidic strength of cationic Al species located in low field was stronger than that of [Al_2_Cl_7_]^−^.

**Fig. 1:**
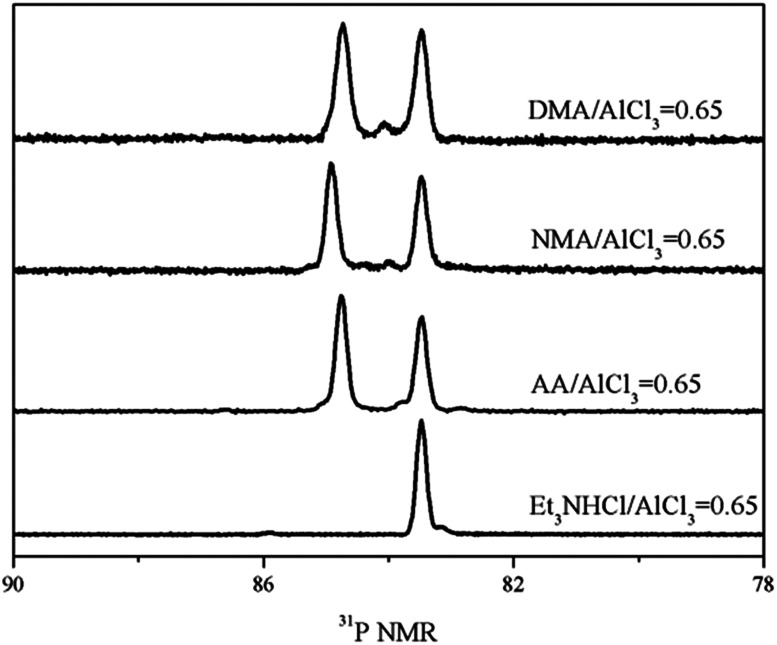
^31^P NMR spectra of three amide–AlCl_3_ based IL analogues and Et_3_NHCl–AlCl_3_ IL with 1 mol% TEPO (ligand/AlCl_3_ molar ratio was 0.65).

Subsequently, *in situ* IR titration method was used to measure the acidic mount of two Lewis acids in neat 0.65NMA–1.0AlCl_3_. The principle of this method is based on the online monitoring of the variation in the characteristic peaks formed by the coordination of indicator (nitrobenzene) with 0.65NMA–1.0AlCl_3_.^22,23^ A quantitative measurement of the acidic amount of 0.65NMA–1.0AlCl_3_ was made based on the typical procedure. 0.65NMA–1.0AlCl_3_ (10 g) was placed into a 25 mL two-necked flask equipped with a stirrer. The silicon probe of the *in situ* IR apparatus was inserted into the 0.65NMA–1.0AlCl_3_, and then the data on the IR spectra were collected. Next, nitrobenzene (0.25 g) was added dropwise to the flask and IR spectra were collected continuously until the absorbance of the characteristic peaks remained constant, meanwhile, the peaks of nitrobenzene itself were observed. The aforementioned steps were repeated until the absorbance of the characteristic peaks did not change with the addition of nitrobenzene. This point was marked as the terminal point of titration, the total mass of nitrobenzene added into 0.65NMA–1.0AlCl_3_ was collected.

As a premise of the *in situ* IR titration method, the characteristic peak formed by the coordination of nitrobenzene with 0.65NMA–1.0AlCl_3_ and the peak of nitrobenzene itself needed to be marked. Fig. 2 shows the IR spectra of neat nitrobenzene, neat 0.65NMA–1.0AlCl_3_, and the mixture of 0.65NMA–1.0AlCl_3_ with nitrobenzene. Two peaks at 1520 and 1346 cm^−1^ were observed in neat nitrobenzene, which were assigned to the *υ*_as_(O–N–O) and *υ*_s_(O–N–O) stretching vibration of –NO_2_ group, respectively.^24,25^ A new peak at 1260 cm^−1^ was observed in the mixture of 0.65NMA–1.0AlCl_3_ with nitrobenzene, which should be assigned to the coordination of nitrobenzene with Lewis acids. Meanwhile, the *υ*_as_(O–N–O) stretching vibration at 1520 cm^−1^ shifted to higher wavenumber 1537 cm^−1^. The *υ*_s_(O–N–O) stretching vibration at 1346 cm^−1^ appeared only in the case that excess nitrobenzene were added into 0.65NMA–1.0AlCl_3_. Therefore, the peaks at 1260 and 1346 cm^−1^ were chosen as the characteristic peaks to observe in the following *in situ* IR titration method.

**Fig. 2:**
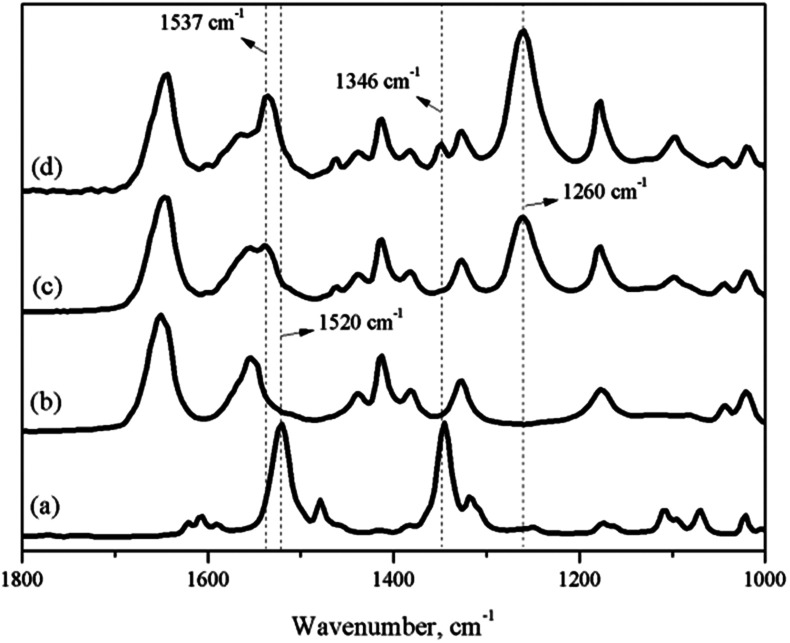
IR spectra of (a) pure nitrobenzene; (b) neat 0.65NMA–1.0AlCl_3_ based IL analogue; (c) nitrobenzene + 0.65NMA–1.0AlCl_3_ based IL analogue (1 : 10 by mass ratio); (d) nitrobenzene + 0.65NMA–1.0AlCl_3_ based IL analogue (1 : 4 by mass ratio).

Fig. 3 shows the variation of the characteristic peaks at 1260 cm^−1^ and 1346 cm^−1^ from the coordination of nitrobenzene with 0.65NMA–1.0AlCl_3_ and *υ*_s_(O–N–O) stretching vibration of nitrobenzene, respectively. A surface plot was generated during the continuous addition of nitrobenzene into 0.65NMA–1.0AlCl_3_ (Fig. 4). The absorbance of the peak at 1260 cm^−1^ increased with the increasing addition of nitrobenzene, while the absorbance of the peak at 1346 cm^−1^ remained almost constant before the terminal point, which attributed that the Lewis acidic amount of 0.65NMA–1.0AlCl_3_ was continuously consumed by nitrobenzene. When the Lewis acidic amount of 0.65NMA–1.0AlCl_3_ was used up, the absorbance of the peak at 1346 cm^−1^ had a significantly increase with the addition of nitrobenzene. The total mass of nitrobenzene from start to terminal point was recorded, and “the molar consumption of nitrobenzene per 1000 g IL analogue” was defined as “activity index” to evaluate the acidic amount of 0.65NMA–1.0AlCl_3_.^26,27^

**Fig. 3:**
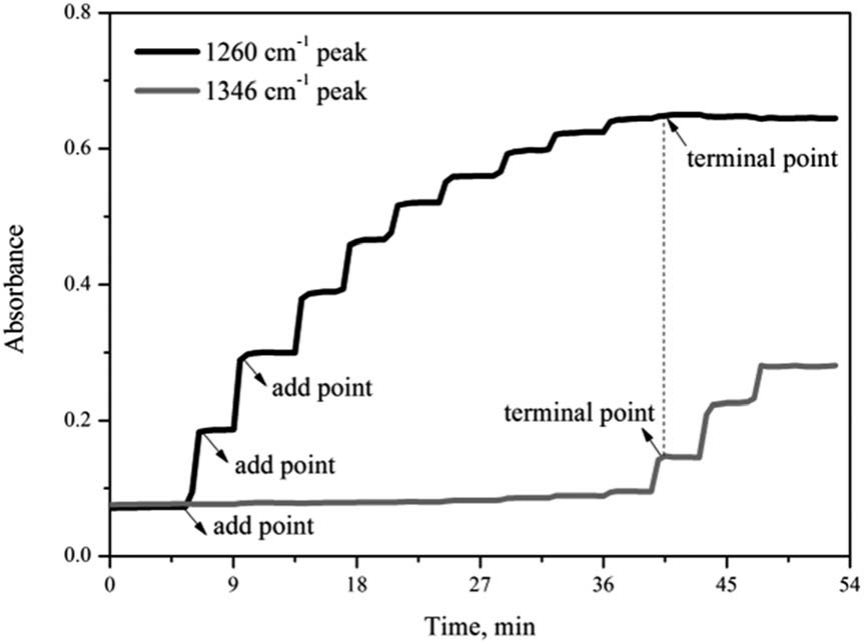
Trend of the characteristic peaks at 1260 cm^−1^ and 1346 cm^−1^ for the addition of nitrobenzene into 0.65NMA–1.0AlCl_3_ based IL analogue.

**Fig. 4:**
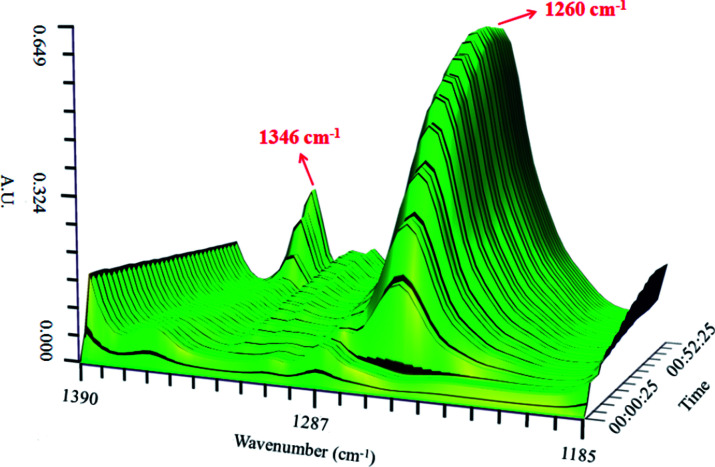
Surface plot in the 1390–1185 cm^−1^ range for the 0.65NMA–1.0AlCl_3_ based IL analogue with the addition of nitrobenzene.

The Lewis acidic amount of several amide–AlCl_3_ based IL analogues with different amide structures and amide/AlCl_3_ molar ratios were measured by *in situ* IR titration method, as shown in Fig. 5. The amide structure affected the Lewis acidic amount of amide–AlCl_3_ based IL analogues,^28^ for example, the Lewis acidic amount of amide–AlCl_3_ based IL analogues (NMA–AlCl_3_ and DMA–AlCl_3_) with bidentate coordination was higher than that of amide–AlCl_3_ based IL analogues (AA–AlCl_3_ and Ur–AlCl_3_) with monodentate coordination under the same amide/AlCl_3_ molar ratio.^29^ This phenomenon was attributed to the fact that the bidentate coordination was more favorable to the asymmetric splitting of AlCl_3_ than the monodentate coordination with the same amide/AlCl_3_ molar ratio, resulting in the more active Lewis species (anionic Al species and cationic Al species). On the other hand, the amide/AlCl_3_ molar ratio also affected the Lewis acidic amount of amide–AlCl_3_ based IL analogues. The Lewis acidic amount of amide–AlCl_3_ based IL analogues increased with the decreasing amide/AlCl_3_ molar ratio. For amide–AlCl_3_ based IL analogues, the balance between neutral molecular Al species and ionic Al species was readily broken with the change of amide/AlCl_3_ molar ratio. The asymmetric splitting degree of Al_2_Cl_6_ increased and the molecular species transformed into ionic species as the amide/AlCl_3_ molar ratio decreased, so the Lewis acidic amount of amide–AlCl_3_ based IL analogue also increased.

**Fig. 5:**
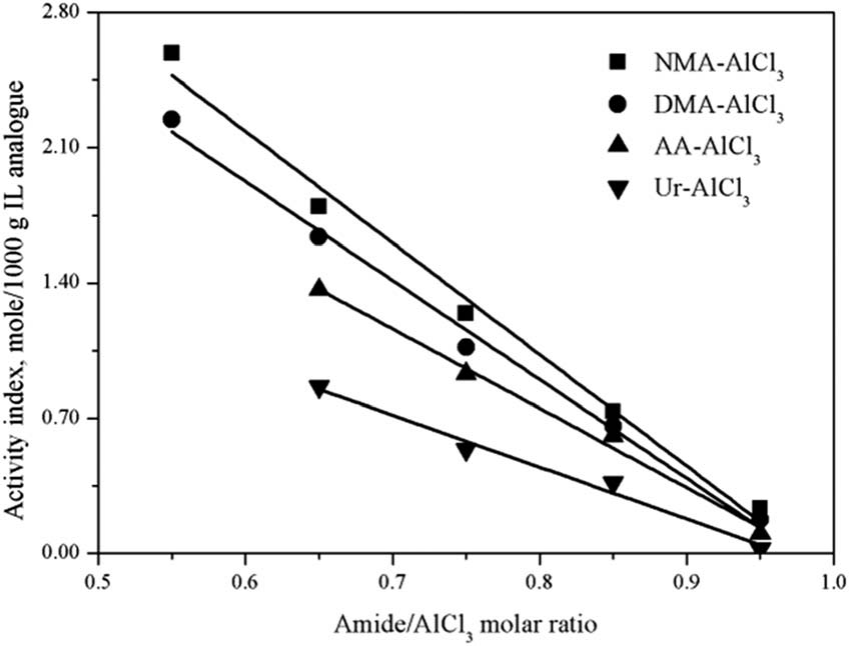
Activity index of four amide–AlCl_3_ based IL analogues at different amide/AlCl_3_ molar ratios.

The total Lewis acidic amount of amide–AlCl_3_ based IL analogues could be measured by *in situ* IR titration method, but the acidic amount of anionic Al species and cationic Al species needed to be further determined. ^27^Al NMR is a good tool to distinguish these Al species, and the peaks at 102.75, 89.30 and 77.05 ppm should be assigned to the anionic Al species ([Al_2_Cl_7_]^−^ and [AlCl_4_]^−^), molecular Al species [AlCl_3_L_*n*_], and cationic Al species [AlCl_2_L_*n*_]^+^, respectively.^29^ The integral area ratio of anionic Al species ([Al_2_Cl_7_]^−^, [AlCl_4_]^−^) to cationic Al species ([AlCl_2_L_*n*_]^+^) was obtained by the normalization method of the peak areas, as shown in Fig. 6. The integral area represented the number of Al nucleus (note: [Al_2_Cl_7_]^−^ had two Al nuclei). Therefore, the integral area ratio of anionic Al species to cationic Al species represented the molar ratio of 2 × [Al_2_Cl_7_]^−^ + [AlCl_4_]^−^ to [AlCl_2_L_*n*_]^+^. The mole of [Al_2_Cl_7_]^−^ + [AlCl_4_]^−^ was equal to that of [AlCl_2_L_*n*_]^+^ according to the conservation law of charge, so the molar ratio of [Al_2_Cl_7_]^−^ to [AlCl_2_L_*n*_]^+^ could be calculated. Taking NMA–AlCl_3_ based IL analogue with different NMA/AlCl_3_ molar ratios as an example, the acidic amount of two Lewis acids ([Al_2_Cl_7_]^−^ and [AlCl_2_L_*n*_]^+^) in NMA–AlCl_3_ based IL analogue was calculated from the results of both *in situ* IR titration and ^27^Al NMR analysis, as listed in Table 1.

**Fig. 6:**
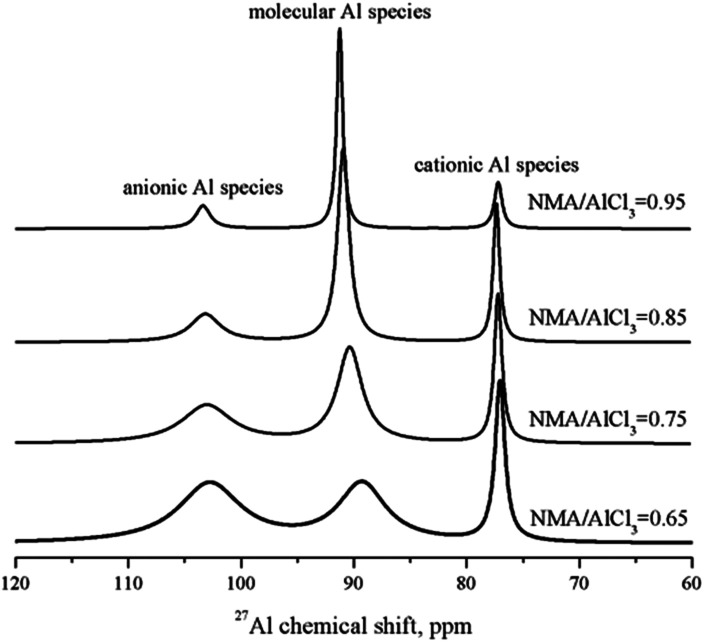
^27^Al NMR spectra of the NMA–AlCl_3_ based IL analogue with different NMA/AlCl_3_ molar ratios.

**Table 1:** Molar ratio of 2 × [Al_2_Cl_7_]^−^ + [AlCl_4_]^−^, [Al_2_Cl_7_]^−^, and [AlCl_4_]^−^ to [AlCl_2_L_*n*_]^+^; and acidic amount of two Lewis acids ([Al_2_Cl_7_]^−^ and [AlCl_2_L_*n*_]^+^) in NMA–AlCl_3_ based IL analogue with different NMA/AlCl_3_ molar ratios

NMA/AlCl_3_	Molar ratio, mol/mol			Acidic amount, mol nitrobenzene/1000 g IL analogue	
	(2 × [Al_2_Cl_7_]^−^ + [AlCl_4_]^−^)/[AlCl_2_L_*n*_]^+^	[Al_2_Cl_7_]^−^/[AlCl_2_L_*n*_]^+^	[AlCl_4_]^−^/[AlCl_2_L_*n*_]^+^	[Al_2_Cl_7_]^−^	[AlCl_2_L_*n*_]^+^
0.65	1.77	0.77	0.23	0.7808	1.0140
0.75	1.58	0.58	0.42	0.4562	0.7866
0.85	1.41	0.41	0.59	0.2134	0.5205
0.95	1.19	0.19	0.81	0.0372	0.1958

## Conclusions

An efficient method to determine the acidic strength and acidic amount of each Lewis acid in amide–AlCl_3_ based IL analogue was proposed in this study. The ^31^P NMR using triethylphosphineoxide as a molecular probe showed that two active Lewis acids ([AlCl_2_L_*n*_]^+^, [Al_2_Cl_7_]^−^) existed in amide–AlCl_3_ based IL analogues, and the acidic strength of [AlCl_2_L_*n*_]^+^ was stronger than that of [Al_2_Cl_7_]^−^. The principle of the *in situ* IR titration method was described in detail, and the total Lewis acidic amount of these IL analogues was measured with nitrobenzene as indicator. The results indicated that the total Lewis acidic amount of amide–AlCl_3_ based IL analogues was related with both the amide structure and the amide/AlCl_3_ molar ratio. The amide–AlCl_3_ based IL analogues with bidentate coordination structure and low amide/AlCl_3_ molar ratio had a high Lewis acidic amount. The Lewis acidic amount of each Lewis acid was calculated further by combining ^27^Al NMR analysis with *in situ* IR titration.

## Experiments

The general route for the synthesis of 0.65amide–1.0AlCl_3_ IL analogue was as follows: anhydrous AlCl_3_ (0.2 mol) was placed in 250 mL two-necked flask; then, amide (0.13 mol; *N*-methylacetamide, NMA; *N*,*N*-dimethylacetamide, DMA; acetamide, AA; urea, Ur) was added slowly while stirring for 30 min. The mixture was then heated to 80 °C and maintained at that temperature until all solids “dissolved” (approximately 4 h).^30^

IR spectra over the 4000 cm^−1^ to 650 cm^−1^ frequency range were obtained at room temperature and at 8 cm^−1^ resolution using an *in situ* IR spectrometer (Mettler-Toledo) equipped with an attenuated total reflectance based silicon probe and a liquid nitrogen-cooled mercury-cadmium-tellurium (MCT) detector. IL analogue (10 g) was placed into a 25 mL two-necked flask equipped with a stirring bar at room temperature. The silicon probe was then inserted into the IL analogue, after which date on the IR spectra were collected. Next, the indicator (0.05 g) was added dropwise to the flask and IR spectra were collected continuously until the characteristic peaks remained constant. The aforementioned steps were repeated. During the measurement, the optical path of the spectrometer was continuously purged with dry N_2_ at a flow rate of 2 mL min^−1^ to eliminate moisture and CO_2_. ^27^Al and ^31^P NMR spectra were obtained using a Bruker Avance spectrometer.

The samples were placed into a 10 mm standard tube by inserting a well-centered capillary. Thereafter, the NMR tube was capped and sealed with parafilm. The aqueous solutions of Al(NO_3_)_3_ (1.0 mol L^−1^) and H_3_PO_5_ (85 wt%) in the capillary was used as an external reference for the ^27^Al NMR and ^31^P NMR chemical shift, respectively. Peak intensities and areas were carefully measured using the Brucker-NMR software package.

## Conflicts of interest

There are no conflicts to declare.

## Supplementary Material
